# Serum urate levels and neurodegenerative outcomes: a prospective cohort study and mendelian randomization analysis of the UK Biobank

**DOI:** 10.1186/s13195-024-01476-x

**Published:** 2024-05-11

**Authors:** Tingjing Zhang, Yu An, Zhenfei Shen, Honghao Yang, Jinguo Jiang, Liangkai Chen, Yanhui Lu, Yang Xia

**Affiliations:** 1 School of Public Health, Wannan Medical College, Wuhu, China; 2 Institutes of Brain Science, Wannan Medical College, Wuhu, China; 3 Department of Endocrinology, Beijing Chao-Yang Hospital, Capital Medical University, Beijing, China; 4 Department of Clinical Nutrition, Yijishan Hospital of Wannan Medical College, Wuhu, China; 5 Department of Clinical Epidemiology, Shengjing Hospital of China Medical University, No. 36, San Hao Street, Shenyang, Liaoning 110004 China; 6 Liaoning Key Laboratory of Precision Medical Research on Major Chronic Disease, Shenyang, China; 7 Department of Nutrition and Food Hygiene, Hubei Key Laboratory of Food Nutrition and Safety, School of Public Health, Tongji Medical College, Huazhong University of Science and Technology, Wuhan, China; 8 School of Nursing, Peking University, No. 38 Xueyuan Rd, Haidian District, Beijing, 100191 China

**Keywords:** Urate, Alzheimer’s disease, Dementia, Parkinson’s disease, Neurodegenerative-related deaths, Prospective cohort study, Mendelian randomization

## Abstract

**Background:**

Previous studies on the associations between serum urate levels and neurodegenerative outcomes have yielded inconclusive results, and the causality remains unclear. This study aimed to investigate whether urate levels are associated with the risks of Alzheimer’s disease and related dementias (ADRD), Parkinson’s disease (PD), and neurodegenerative deaths.

**Methods:**

This prospective study included 382,182 participants (45.7% men) from the UK Biobank cohort. Cox proportional hazards models were used to assess the associations between urate levels and risk of neurodegenerative outcomes. In the Mendelian randomization (MR) analysis, urate-related single-nucleotide polymorphisms were identified through a genome-wide association study. Both linear and non-linear MR approaches were utilized to investigate the potential causal associations.

**Results:**

During a median follow-up period of 12 years, we documented 5,400 ADRD cases, 2,553 PD cases, and 1,531 neurodegenerative deaths. Observational data revealed that a higher urate level was associated with a decreased risk of ADRD (hazard ratio [HR]: 0.93, 95% confidence interval [CI]: 0.90, 0.96), PD (HR: 0.87, 95% CI: 0.82, 0.91), and neurodegenerative death (HR: 0.88, 95% CI: 0.83, 0.94). Negative linear associations between urate levels and neurodegenerative events were observed (all *P*-values for overall < 0.001 and all *P*-values for non-linearity > 0.05). However, MR analyses yielded no evidence of either linear or non-linear associations between genetically predicted urate levels and the risk of the aforementioned neurodegenerative events.

**Conclusion:**

Although the prospective cohort study demonstrated that elevated urate levels were associated with a reduced risk of neurodegenerative outcomes, MR analyses found no evidence of causality.

**Supplementary Information:**

The online version contains supplementary material available at 10.1186/s13195-024-01476-x.

## Background

Neurological disorders rank foremost in causing disability and stand as the second most common cause of death worldwide, accounting for 11.6% of global disability-adjusted life-years and 16.5% of all deaths [1]. Globally, Alzheimer’s disease and related dementias (ADRD) and Parkinson’s disease (PD) are the most prevalent neurodegenerative diseases [1, 2]. Currently, there are more than 55 million individuals with ADRD, as well as more than 8.5 million individuals with PD worldwide [3, 4]. The economic burden of ADRD on the global economy amounts to 1.3 trillion US dollars, with nearly 10 million new cases reported each year [3]. PD has resulted in 5.8 million disability-adjusted life years, reflecting an 81% increase since 2000 [4]. At present, neither ADRD nor PD has a cure, emphasizing the importance of identifying and focusing on modifiable risk factors associated with these conditions.

Urate, the final product of human purine metabolism, serves as a potent antioxidant [5, 6]. It plays a significant role in human physiology by contributing to approximately 60% of the scavenging activity against free radicals [7]. Urate plays a crucial role in neutralizing and eliminating reactive oxygen species, thereby protecting cells and tissues from oxidative damage [8]. The antioxidant properties of urate are crucial for maintaining cell function and preventing conditions associated with oxidative stress [9, 10]. Additionally, these antioxidant properties have led to suggestions that urate may be a neuroprotective agent [7, 11]. However, while the associations of urate levels with neurodegenerative diseases have been explored, the findings are inconsistent and conflicting [12–15]. This inconsistency may be attributed to potential confounding factors and possible reverse causation influencing the observed associations. Furthermore, it remains unclear whether the association between urate levels and risk of neurodegenerative outcomes is causal.

Mendelian randomization (MR) is an approach of epidemiological studies that uses genetic variants associated with exposure as instrumental variables to establish causal effects on outcomes [16]. The MR design eliminates the impact of confounding factors as alleles are randomly allocated during gamete formation and conception [17]. Consequently, the results of MR avoid the bias of reverse causation and confounding factors [18].

Therefore, we aimed to determine the associations between urate levels and risk of neurodegenerative diseases, especially ADRD, PD, and neurodegenerative death, based on a large prospective population-based observational analysis and the MR approach, and to provide a stronger scientific basis to enhance the efficacy of health management strategies.

## Materials and methods

### Study populations

UK Biobank is a prospective study that enrolled more than 500,000 individuals aged 40 to 79 years from 22 evaluation centers across the United Kingdom between April 2006 to December 2010. During recruitment, all participants were assessed for demographic information, lifestyle factors, bodily measurements, and other health-related parameters by trained health professionals. Additionally, blood specimens were collected for genotyping. The UK Biobank study protocol is publicly available at https://www.ukbiobank.ac.uk/.

In this large population-based study of 502,461 participants, several exclusion criteria were applied to ensure data quality: (1) individuals with prevalent ADRD or PD at baseline; (2) those with missing data on urate levels, genetic information, and related covariates; (3) individuals with sex discordance; (4) outliers with genotype missingness or heterozygosity; (5) individuals with genetic kinship to other participants; and (6) individuals of non-European ancestry. As a result, a final sample of 382,182 participants was retained for the analysis. The flowchart is shown in Fig. S1.

The UK Biobank study was approved by the Northwest Multi-Center Research Ethics Committee, and each participant provided written informed consent before participating in the study. The data resource used for this study was obtained under application number 63,454 from the UK Biobank.

### Assessment of exposure, outcome, and covariates

Baseline serum urate levels were measured using the uricase pedigree analysis package of the Beckman Coulter AU5800 platform (Randox Biosciences, Crumlin, UK). Participants were categorized into quartiles based on the distribution of urate levels according to sex. “Quartile 1” refers to the lowest 25% of participants with the lowest urate level, while “quartile 4” represents the highest 25% of participants with the highest urate level.

Neurodegenerative outcomes were identified using data on admissions and diagnoses with primary or secondary diagnosis based on the International Classification of Diseases (detailed information provided in Table S1) [19, 20]. The follow-up period ranged from March 16, 2006 to the end endpoint of follow-up (September 30, 2021 for centers in England; February 28, 2018, for centers in Wales; and July 31, 2021, for centers in Scotland). Person-years were calculated for each participant from the date of baseline assessment to the occurrence of neurodegenerative outcomes, death, or the end of follow-up, whichever occurred first.

Covariates possibly affecting the associations between urate levels and neurodegenerative outcomes, as indicated by previous studies, were taken into account in our analysis. A baseline touch-screen questionnaire was used to assess the potential confounding variables, including sociodemographic and lifestyle factors (e.g., age, sex, educational levels, smoking status, alcohol consumption and dietary habits), as well as personal and family history of diseases. Based on the baseline food frequency questionnaire, a diet score was calculated using the following elements: vegetables, fruits, fish, processed meat, unprocessed red meat, whole grains, and refined grains, as conducted in previous studies [21, 22]. Each diet factor received 1 point: consumption of at least 3 servings of vegetables per day, at least 3 servings of fruit per day, at least 2 servings of fish per week, no more than 1 serving of processed meat per week, no more than 1.5 servings of unprocessed red meat per week, at least 3 servings of whole grains per day, and no more than 1.5 servings of refined grains per week. The total diet score ranged from 0 to 7. Details of covariates were provided in Table S3.

### Genetic instrument for urate

The genotyping procedure and DNA array used in the UK Biobank study have been previously described [23]. In brief, each participant’s blood specimen was genotyped using the custom Affymetrix UK Biobank Axiom array. The genotyping data underwent phasing and imputation; SHAPEIT3 was used for phasing and IMPUTE3 was used for imputation, with a merged reference panel of UK10K and 1000 Genomes Phase 3 [24].

We used 20 independent single nucleotide polymorphisms (SNPs) (*P* < 5 × 10^− 8^, r^2^ < 0.1 within a 1000 kb window) identified in a genome-wide association analysis as genetic instruments in the MR (Table S2) [25]. These SNPs were used to construct the genetic risk score (GRS). The calculation of the GRS for each SNP involved coding them as 0, 1, or 2 based on the number of risk alleles, and each SNP was weighted by its relative effect size (β coefficient). The GRS for each individual was then obtained by summing the weighted scores using the PLINK “–score” command and the z-standardized value. The distribution of urate-related GRS is shown in Fig. S2. In this study, the genetic instrument showed a strong association with urate levels, with an F statistic of 173 and a *P*-value < 0.0001.

### Statistical analysis

Baseline characteristics of the study population were outlined across quartiles of the urate levels, with continuous variables expressed as mean (standard deviation, SD) and categorical variables as percentages (%). Cox proportional hazard regression models were used to examine the associations of urate levels with neurodegenerative outcomes. Proportional hazards were tested using scaled Schoenfeld’s residuals. Three models were established: (1) model 1 adjusted for age, sex, and body mass index (BMI); (2) model 2 additionally adjusted for education levels, Townsend deprivation index, smoking status, and drinking status based on model 1; and (3) model 3 additionally adjusted for family history of diseases (hypertension, cardiovascular disease, and diabetes), healthy diet score, and personal history of diseases (kidney disease, hypertension, cardiovascular disease, and diabetes) based on model 2. The *P*-value for trend was calculated using the median value of urate in each quartile as a continuous variable [26]. Restricted cubic splines based on Cox proportional hazards regression model [27] were used to evaluate non-linear associations between urate levels and neurodegenerative outcomes in the multivariable model with 3 knots at the 25th, 50th, and 75th percentiles of the urate levels (with the minimum value used as the reference). To strengthen the robustness of the results, we performed several sensitivity analyses as follows: (1) excluded participants who had incident neurodegenerative outcomes at the initial 5 follow-up years to avoid reverse causality; (2) repeated the analysis after stratifying by age, sex, and BMI; (3) conducted Fine–Gray competing risk analysis to assess the competitive risk of non-neurodegenerative death [28]; and (4) divided the neurodegenerative death into deaths due to ADRD and PD respectively.

We employed both linear and non-linear MR methods to assess potential causal associations between urate levels and neurodegenerative outcomes. For the linear MR analyses, we examined the associations between urate-related GRS and neurodegenerative outcomes using a Cox regression model. The model was adjusted for various covariates, including age, sex, BMI, educational levels, Townsend deprivation index, smoking status, alcohol consumption, family history of diseases (hypertension, cardiovascular disease, and diabetes), healthy diet score, personal history of diseases (kidney disease, hypertension, cardiovascular disease, and diabetes), the first 10 principal components of ancestry, and genotype measurement batch. In the sensitivity analyses, (1) we employed an unweighted GRS model, calculated by summing the number of urate-related increasing alleles; (2) the SNP rs2231142, identified as the strongest in previous GWAS, was used as an instrumental variable to mitigate the potential introduction of horizontal pleiotropy [25]; and (3) the urate-related GRS was divided into quartiles to assess the linear MR results. In the non-linear MR analyses, we divided the sample into five strata based on the residual urate levels, which represented the differential value between the observed urate level and the genetically predicted urate level. Within each stratum, we evaluated the linear MR estimate, which contributed to the localized average causal effect (LACE) [29]. A meta-regression of LACE estimates against the mean of the exposure in each stratum was performed using a flexible semiparametric framework that applied the derivative of fractional polynomial models. This assessment aimed to determine whether a non-linear model offered a better fit for the LACE estimates compared to a linear model [30]. Two tests for non-linearity were conducted as follows: (1) a Cochran’s Q statistic to assess heterogeneity by analyzing differences between the LACE estimates, and (2) a trend test that involved meta-regression of LACE estimates against the mean value of urate in each stratum.

*P-*values were two-sided with < 0.05 defined as statistically significant. Statistical Analysis System 9.4 software for Windows was used to conduct the cohort analyses (SAS Institute Inc., Gary, NC, USA), and MR analyses were performed using R version 4.2.3 with “*TwoSampleMR*” and “*NLMR*” packages.

## Results

### Baseline characteristics of the study population

In this study, a total of 382,182 participants (174,990 [45.7%] men and 207,192 [54.2%] women) were included. Over a median follow-up period of 12 years, 5,400 ADRD cases, 2,553 PD cases, and 1,531 neurodegenerative deaths were documented. Table 1 presents the baseline characteristics categorized by urate levels. Participants with elevated urate levels tended to be older and more frequently drinkers. They also possessed higher BMI values and showed a greater propensity for medical histories of hypertension, diabetes, kidney disease, and cardiovascular disease. Conversely, they scored lower in healthy diet, and educational level compared to those with reduced urate levels.

**Table 1:** Participants baseline characteristics according to serum urate levels in the UK Biobank (*n* = 382,182)

Characteristics	Serum urate levels				*P* value ^1^
	Level 1	Level 2	Level 3	Level 4	
NO.	95,603	95,575	95,436	95,568	
**Demographics**					
Age (years)	55.7 (8.2) ^1^	56.4 (8.0)	57.1 (7.8)	58.1 (7.5)	**< 0.0001**
Sex (male, %)	45.7 ^2^	45.8	45.7	45.7	0.99
Education (≥ College graduate, %)	33.9	32.7	30.6	26.7	**< 0.0001**
Townsend Deprivation Index	-1.6 (2.9)	-1.6 (2.8)	-1.6 (2.8)	-1.4 (2.9)	**< 0.0001**
**Lifestyle factors**					
Smoking status					
Never	57.6	56.3	54.3	50.5	**< 0.0001**
Previous	30.7	33.6	36.4	40.7	**< 0.0001**
Current	11.7	10.2	9.4	8.8	**< 0.0001**
Alcohol consumption					
≥ Once per day	18.4	20.6	22.1	24.0	**< 0.0001**
≥ Once per week	50.7	51.8	51.5	48.7	**< 0.0001**
≥ Once per month	12.1	11.4	10.8	10.1	**< 0.0001**
< Once per month	11.3	10.1	9.8	10.9	**< 0.0001**
Never	7.6	6.1	5.8	6.4	**< 0.0001**
Healthy diet score					
0	0.9	0.8	0.9	0.9	0.08
1	4.7	4.8	5.0	5.6	**< 0.0001**
2	12.7	13.0	13.7	15.0	**< 0.0001**
3	22.0	22.7	23.6	24.5	**< 0.0001**
4	26.9	27.1	26.9	26.7	0.23
5	22.0	21.3	20.3	18.7	**< 0.0001**
6	9.5	9.2	8.6	7.8	**< 0.0001**
7	1.3	1.0	1.0	0.9	**< 0.0001**
**Clinical and laboratory measures**					
Urate (umol/L)	228.6 (45.5)	282.9 (43.2)	325.3 (45.8)	399.6 (63.5)	**< 0.0001**
Body mass index (kg/m^2^)	25.3 (3.9)	26.5 (4.1)	27.7 (4.4)	29.9 (5.1)	**< 0.0001**
**Family diseases history**					
Cardiovascular disease (yes, %)	54.8	56.4	57.7	60.1	**< 0.0001**
Hypertension (yes, %)	46.0	46.7	47.2	48.8	**< 0.0001**
Diabetes (yes, %)	18.3	19.5	20.8	22.8	**< 0.0001**
**Medical history**					
Hypertension (yes, %)	18.1	21.1	26.2	40.5	**< 0.0001**
Diabetes (yes, %)	5.0	3.5	3.8	6.1	**< 0.0001**
Kidney disease (yes, %)	1.5	1.6	1.7	2.2	**< 0.0001**
Cardiovascular disease (yes, %)	4.8	5.0	5.7	8.1	**< 0.0001**

^1^ Analysis of variance or chis-square test where appropriate

^2^ Mean (standard deviation) (all such values)

^3^ Percentage (all such values)

### Observational findings

**Fig. 1:**
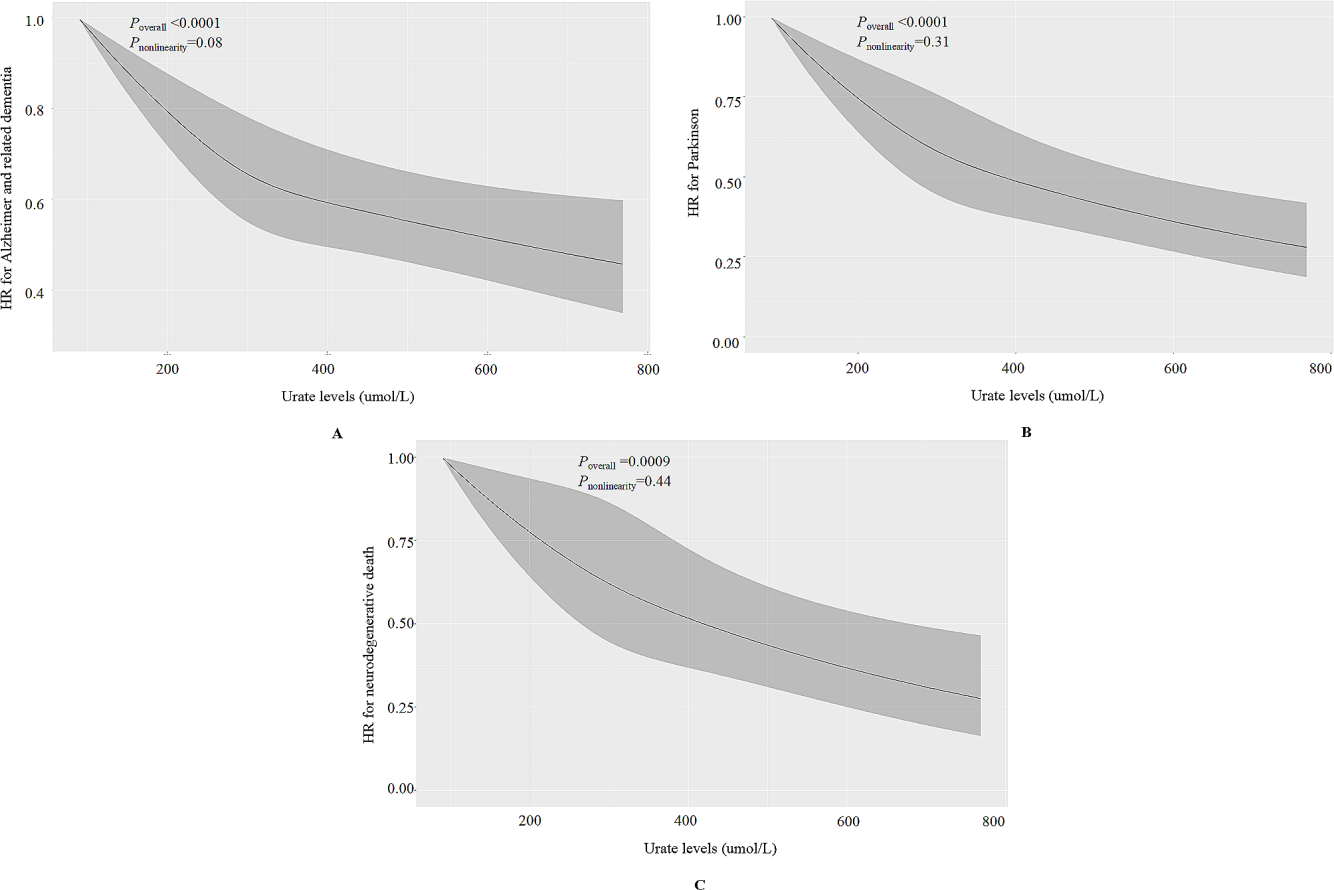
Shape of the association between urate and neurodegenerative outcomes using restricted cubic spline based on observational data. Adjusted for age, sex, BMI, education levels, Townsend deprivation index, smoking status, alcohol consumption, family history of diseases (hypertension, cardiovascular disease, and diabetes), healthy diet score, and history of diseases (kidney disease, hypertension, cardiovascular disease, and diabetes)

Table 2 shows the associations between urate levels and risk of neurodegenerative outcomes. In the cohort analyses, urate levels exhibited inverse associations with the risk of ADRD, PD, and neurodegenerative death. With each increase of one SD in urate levels, the risk of ADRD, PD, and neurodegenerative death decreased by 7% (HR: 0.93, 95% CI: 0.90, 0.96), 13% (HR: 0.87, 95% CI: 0.82, 0.91), and 12% (HR: 0.88, 95% CI: 0.83, 0.94), respectively. The restricted cubic spline curves demonstrated that there was no non-linear association between urate levels and ADRD (*P*-value for overall < 0.0001, *P*-value for non-linearity = 0.08), PD (*P*-value for overall < 0.0001, *P*-value for non-linearity = 0.31), and neurodegenerative death (*P*-value for overall = 0.0009, *P*-value for non-linearity = 0.44) (Fig. 1). In sensitivity analyses, we achieved consistent findings when: (1) excluding participants with incident neurodegenerative outcomes within the initial 5 follow-up years (Table S4); (2) conducting subgroup analyses stratified by age, sex, and BMI (Table S5); (3) using a competing risk regression model for the analyses (Table S6); (4) divided the neurodegenerative death into deaths due to ADRD and PD respectively (Table S7).

**Table 2:** Association between urate levels and risk of neurodegenerative outcomes

	Quartiles of urate levels				*P* for trend ^1^	Per SD increase	*P* values^1^
	Quartile 1	Quartile 2	Quartile 3	Quartile 4			
Level of urate (umol/L): median	219.0	260.9	304.7	403.5		302.9	
Number of participants	95,603	95,575	95,436	95,568		382,182	
**Alzheimer and related dementia**							
Number of cases	1,390	1,251	1,228	1,531		5,400	
Person years	1,107,735	1,110,503	1,108,912	1,106,313		4,433,463	
Model 1 ^2^	1 (reference)	**0.82 (0.76, 0.89)** ^3^	**0.74 (0.68, 0.80)**	**0.80 (0.74, 0.87)**	**< 0.0001**	**0.92 (0.89, 0.95)**	**< 0.0001**
Model 2 ^4^	1 (reference)	**0.84 (0.78, 0.91)**	**0.77 (0.71, 0.83)**	**0.84 (0.78, 0.91)**	**< 0.0001**	**0.93 (0.90, 0.97)**	**0.0001**
Model 3 ^5^	1 (reference)	**0.87 (0.80, 0.94)**	**0.79 (0.73, 0.86)**	**0.83 (0.77, 0.90)**	**< 0.0001**	**0.93 (0.90, 0.96)**	**< 0.0001**
**Parkinson**							
Number of cases	709	631	609	604		2,553	
Person years	1,108,082	1,110,892	1,109,614	1,107,690		4,436,278	
Model 1	1 (reference)	**0.83 (0.75, 0.93)**	**0.76 (0.68, 0.84)**	**0.68 (0.60, 0.76)**	**< 0.0001**	**0.85 (0.81, 0.89)**	**< 0.0001**
Model 2	1 (reference)	**0.85 (0.76, 0.94)**	**0.78 (0.69, 0.87)**	**0.70 (0.62, 0.79)**	**< 0.0001**	**0.86 (0.82, 0.91)**	**< 0.0001**
Model 3	1 (reference)	**0.86 (0.77, 0.95)**	**0.79 (0.70, 0.88)**	**0.71 (0.63, 0.79)**	**< 0.0001**	**0.87 (0.82, 0.91)**	**< 0.0001**
**Neurodegenerative death**							
Number of cases	439	374	351	367		1,531	
Person years	1,110,801	1,113,195	1,111,570	1,109,565		4,445,131	
Model 1	1 (reference)	**0.80 (0.69, 0.92)**	**0.71 (0.62, 0.82)**	**0.68 (0.59, 0.79)**	**< 0.0001**	**0.86 (0.81, 0.91)**	**< 0.0001**
Model 2	1 (reference)	**0.82 (0.71, 0.94)**	**0.74 (0.64, 0.85)**	**0.71 (0.61, 0.82)**	**< 0.0001**	**0.87 (0.82, 0.93)**	**< 0.0001**
Model 3	1 (reference)	**0.84 (0.73, 0.97)**	**0.76 (0.66, 0.88)**	**0.72 (0.62, 0.83)**	**< 0.0001**	**0.88 (0.83, 0.94)**	**< 0.0001**

*Abbreviations* SD, standard deviation

^1^ Analysis by Cox proportional hazards regression models

^2^ Adjusted for age, sex, and BMI

^3^ Hazard ratios (95% confidence interval) (all such values)

^4^ Additionally adjusted for education levels, Townsend deprivation index, smoking status, and alcohol consumption

^5^ Additionally adjusted for family history of diseases (hypertension, cardiovascular disease, and diabetes), healthy diet score, and history of diseases (kidney disease, hypertension, cardiovascular disease, and diabetes)

### Mendelian randomization results

**Fig. 2:**
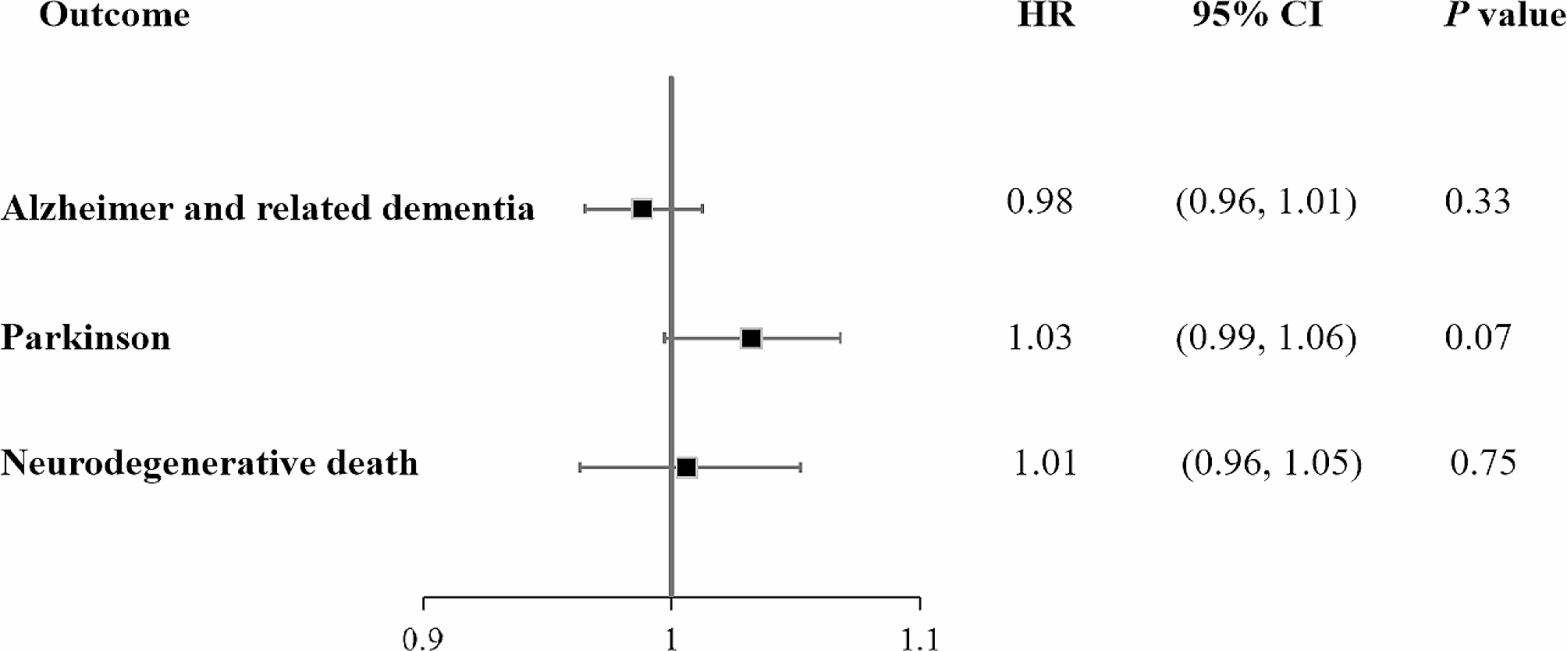
The casual associations between urate levels and neurodegenerative outcomes using linear MR analysis. Adjusted for age, sex, BMI, education levels, Townsend deprivation index, smoking status, alcohol consumption, family history of diseases (hypertension, cardiovascular disease, and diabetes), healthy diet score, history of diseases (kidney disease, hypertension, cardiovascular disease, and diabetes), first 10 principal components of ancestry, and genotype measurement batch

**Fig. 3:**
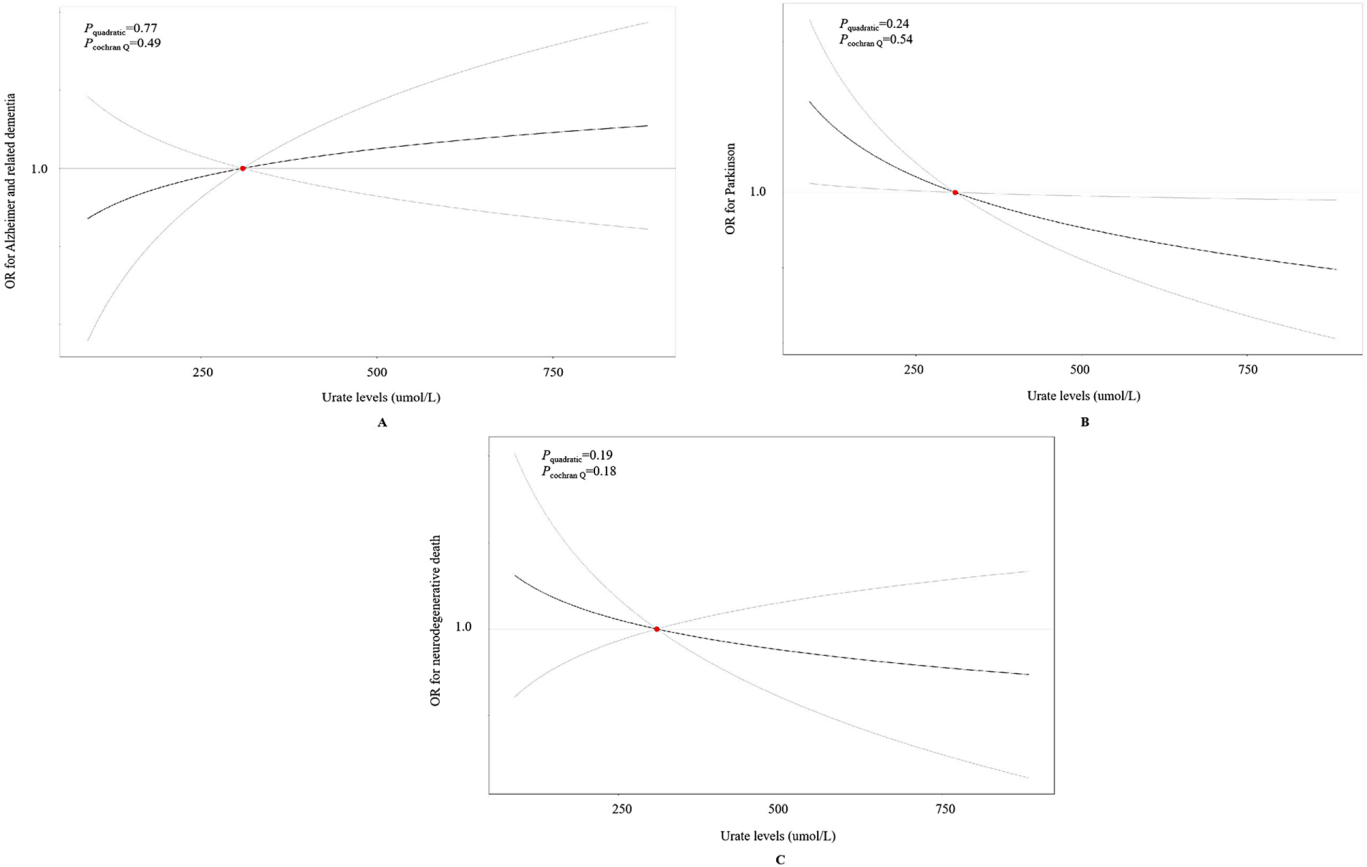
Shape of casual relationship between urate and neurodegenerative outcomes using non-linear MR method. Adjusted for age, sex, BMI, education levels, Townsend deprivation index, smoking status, alcohol consumption, family history of diseases (hypertension, cardiovascular disease, and diabetes), healthy diet score, history of diseases (kidney disease, hypertension, cardiovascular disease, and diabetes), first 10 principal components of ancestry, and genotype measurement batch

As depicted in Fig. 2, there was no linear association between genetically predicted urate levels and risk of ADRD (HR: 0.98, 95% CI: 0.96, 1.01), PD (HR: 1.03, 95% CI: 0.99, 1.06), and neurodegenerative death (HR: 1.01, 95% CI: 0.96, 1.05). Additionally, consistent results were observed in the sensitivity analyses when re-evaluating the associations between unweighted urate-related GRS and neurodegenerative outcomes (Fig. S2), using rs2231142 as an instrument variable (Fig. S3), or dividing the urate-related GRS into quartiles (Table S8). Moreover, there was no evidence of non-linear causal effects between genetically predicted urate levels and risk of ADRD (*P*_quadratic_ = 0.77, *P*_cochran Q_ = 0.49), PD (*P*_quadratic_ = 0.24, *P*_cochran Q_ = 0.54), and neurodegenerative death (*P*_quadratic_ = 0.19, *P*_cochran Q_ = 0.18) (Fig. 3).

## Discussion

We investigated the associations between urate levels and neurodegenerative outcomes using a comprehensive approach that involved a large population-based cohort and complementary MR analyses. Our findings suggest that, while elevated urate levels are associated with a reduced risk of incident neurodegenerative outcomes, both linear and non-linear MR analyses demonstrated no evidence of causality of these associations. These results have clinical significance because of the limited research available on the intricate associations between urate levels and neurodegenerative outcomes.

Previous observational epidemiological studies have explored the associations between urate levels and risk of neurodegenerative outcomes [12, 31–33], which support part of our findings reported herein. Scheepers et al. found that long-term follow-up data from a Sweden perspective study, which spanned 44 years, highlighted the protective role of urate in the development of dementia across subtypes [31]. A meta-analysis of 21 case-control studies and 3 cohort studies indicated a potential inverse association between serum uric acid levels and Alzheimer’s disease (AD) risk [12]. Another systematic review involving 23 studies (5,575 participants) reported low serum uric acid levels as a potential risk factor for both AD and PD [32]. Additionally, a dose-response meta-analysis of 15 studies involving 449,816 participants and 14,687 cases revealed a 6% reduction in PD risk for every 1 mg/dL increase in the urate level [33]. However, a population-based cohort study with a 12-year follow-up period reported inconsistent findings, suggesting that elevated serum uric acid levels were associated with an increased risk of dementia [13]. Based on a large prospective cohort study, we observed a negative association between urate levels and neurodegenerative outcomes. The underlying mechanism may lie in urate’s antioxidant properties, which could offer protection against neurodegeneration by reducing oxidative stress and inflammation [7, 8]. Additionally, experimental models of neurodegenerative diseases have shown that urate has neuroprotective effects [34]. The inconsistency between the results of several studies may be attributed to several factors, including differences in study populations, methodologies, outcome definitions, and potential confounding variables.

To enhance the public health implications of our findings, we also employed MR methods. Although our observational analyses revealed significant negative associations between urate levels and risk of neurodegenerative outcomes in the prospective cohort, the MR analyses did not support a causal association. Through the use of SNPs as exposure proxies, which are randomly distributed among individuals, MR analysis offers an analogous approach to a randomized controlled trial [35]. Consistent with our results, a previous double-blind, placebo-controlled, phase III randomized trial involving 587 individuals did not establish an association between sustained urate-elevating treatment and PD risk [36]. The results of our MR study also suggest that increasing urate levels are unlikely to offer clinical benefits in reducing the risk of neurodegenerative outcomes, including ADRD, PD, and neurodegenerative death. This provides an important public health implication, indicating that elevated urate levels may not be effective for preventing neurodegenerative events.

This is the first large-scale investigation examining the associations between urate levels and ADRD, PD, and neurodegenerative death using complementary analyses (cohort and MR analyses), which increased the reliability of our conclusions. The utilization of a large population-based dataset enhanced the statistical power and the applicability of our findings. Furthermore, our MR analyses employed robust instrumental variables, thereby minimizing the potential for weak instrument bias. Additionally, we rigorously assessed key assumptions, ensuring that primary instruments were not related to potential confounders.

Our study has several limitations. Firstly, the potential for selection bias and residual confounding exists, despite our adjustments for multiple confounders. The potential for confounding by unaccounted factors also exists. Secondly, the MR analysis was constrained by the limited number of SNPs used. Although we included a substantial number of genetic variants, a score encompassing a greater array of urate-related SNPs would enhance the robustness of causal investigation. Additionally, it should be acknowledged that certain SNPs utilized in our analysis may exhibit potential correlations with unidentified factors associated with neurodegenerative outcomes. Consequently, we cannot entirely dismiss the potential influence of pleiotropic effects on our findings. Thirdly, the diagnosis of neurodegenerative events was derived from registry-based data rather than comprehensive neuropsychological assessments. Although registry-based diagnoses generally exhibit good accuracy, the potential for misclassification among certain study participants cannot be entirely ruled out. Finally, it is important to note that the participants in this study predominantly belong to the White British ethnicity, which might limit the generalizability of our findings to other ethnicities or populations.

## Conclusion

Our study revealed significant linear negative associations between urate levels and risk of ADRD, PD, and neurodegenerative death, as evidenced by a comprehensive large-scale prospective cohort study. However, the MR analyses did not sustain the causality aspect, regardless of the application of linear and non-linear MR analyses. This underscores a crucial public health message that elevated urate levels may not be essential for mitigating neurodegenerative outcomes. Nonetheless, additional research is warranted to validate these findings.

### Electronic supplementary material

Below is the link to the electronic supplementary material.

Supplementary Material 1

## Data Availability

Data are available in a public, open access repository. This research has been conducted using the UK Biobank Resource under Application Number 63454. The UK Biobank data are available on application to the UK Biobank (https://www.ukbiobank.ac.uk/).

## Abbreviations

ADRD: Alzheimer’s disease and related dementias
PD: Parkinson’s disease
MR: Mendelian randomization
HR: Hazard ratio
CI: Confidence interval

## References

[1] Collaborators GBDN (2019). Global, regional, and national burden of neurological disorders, 1990–2016: a systematic analysis for the global burden of Disease Study 2016. Lancet Neurol.

[2] (WHO). W.H.O. *Neurological Disorders Fact Sheet*. https://www.who.int/news-room/fact-sheets/detail/neurological-disorders.

[3] (WHO). W.H.O. *Dementia Fact Sheet*. https://www.who.int/news-room/fact-sheets/detail/dementia.

[4] (WHO). W.H.O. *Parkinson Disease Fact Sheet*. https://www.who.int/news-room/fact-sheets/detail/parkinson-disease.

[5] Alvarez-Lario B, Macarron-Vicente J (2010). Uric acid and evolution. Rheumatology (Oxford).

[6] Richette P, Bardin T (2010). Gout Lancet.

[7] Waring WS (2002). Uric acid: an important antioxidant in acute ischaemic stroke. QJM.

[8] Maxwell SR (1997). Antioxidant status in patients with uncomplicated insulin-dependent and non-insulin-dependent diabetes mellitus. Eur J Clin Invest.

[9] Baillie JK (2007). Endogenous urate production augments plasma antioxidant capacity in healthy lowland subjects exposed to high altitude. Chest.

[10] Waring WS (2003). Uric acid reduces exercise-induced oxidative stress in healthy adults. Clin Sci (Lond).

[11] Chen X, Wu G, Schwarzschild MA (2012). Urate in Parkinson’s disease: more than a biomarker?. Curr Neurol Neurosci Rep.

[12] Du N (2016). Inverse Association between serum uric acid levels and Alzheimer’s Disease Risk. Mol Neurobiol.

[13] Latourte A (2018). Uric acid and incident dementia over 12 years of follow-up: a population-based cohort study. Ann Rheum Dis.

[14] Gao X (2016). Prospective study of plasma urate and risk of Parkinson disease in men and women. Neurology.

[15] Kia DA (2018). Mendelian randomization study shows no causal relationship between circulating urate levels and Parkinson’s disease. Ann Neurol.

[16] Davey Smith G, Hemani G (2014). Mendelian randomization: genetic anchors for causal inference in epidemiological studies. Hum Mol Genet.

[17] Smith GD, Ebrahim S (2003). Mendelian randomization’: can genetic epidemiology contribute to understanding environmental determinants of disease?. Int J Epidemiol.

[18] Verduijn M (2010). Mendelian randomization: use of genetics to enable causal inference in observational studies. Nephrol Dial Transpl.

[19] Shang X (2022). Association of a wide range of chronic diseases and apolipoprotein E4 genotype with subsequent risk of dementia in community-dwelling adults: a retrospective cohort study. EClinicalMedicine.

[20] Wilkinson T (2019). Identifying dementia outcomes in UK Biobank: a validation study of primary care, hospital admissions and mortality data. Eur J Epidemiol.

[21] Pazoki R (2018). Genetic predisposition to high blood pressure and lifestyle factors: associations with midlife blood pressure levels and Cardiovascular events. Circulation.

[22] Wang L (2022). Air pollution and risk of chronic obstructed pulmonary disease: the modifying effect of genetic susceptibility and lifestyle. EBioMedicine.

[23] Bycroft C, Petkova FC, Band D, Elliott G, Sharp LT, Motyer K, Vukcevic A, Delaneau D, O’Connell O, Cortes J, Welsh A, McVean S, Les G lie, Donnelly S, Marchini P. J., *Genome-wide genetic data on ~ 500,000 UK Biobank participants* bioRxiv, July 20, 2017.

[24] J M. *UK Biobank. UK Biobank phasing and imputation documentation*

[25] Kottgen A (2013). Genome-wide association analyses identify 18 new loci associated with serum urate concentrations. Nat Genet.

[26] Sotos-Prieto M (2017). Association of Changes in Diet Quality with Total and cause-specific mortality. N Engl J Med.

[27] Desquilbet L, Mariotti F (2010). Dose-response analyses using restricted cubic spline functions in public health research. Stat Med.

[28] Noordzij M (2013). When do we need competing risks methods for survival analysis in nephrology?. Nephrol Dial Transpl.

[29] Staley JR, Burgess S (2017). Semiparametric methods for estimation of a nonlinear exposure-outcome relationship using instrumental variables with application to mendelian randomization. Genet Epidemiol.

[30] Sun YQ (2019). Body mass index and all cause mortality in HUNT and UK Biobank studies: linear and non-linear mendelian randomisation analyses. BMJ.

[31] Scheepers L (2019). Urate and risk of Alzheimer’s disease and vascular dementia: a population-based study. Alzheimers Dement.

[32] Zhou Z (2021). Serum uric acid and the risk of dementia: a systematic review and Meta-analysis. Front Aging Neurosci.

[33] Chang H (2022). Dose-response meta-analysis on urate, gout, and the risk for Parkinson’s disease. NPJ Parkinsons Dis.

[34] Squadrito GL (2000). Reaction of uric acid with peroxynitrite and implications for the mechanism of neuroprotection by uric acid. Arch Biochem Biophys.

[35] Zhou H (2020). Mendelian randomization study showed no causality between metformin use and lung cancer risk. Int J Epidemiol.

[36] Parkinson Study Group (2021). Effect of urate-elevating inosine on early Parkinson Disease Progression: the SURE-PD3 Randomized Clinical Trial. JAMA.

