# Supplementary material for: Serum urate levels and neurodegenerative outcomes: a prospective cohort study and mendelian randomization analysis of the UK Biobank
Source: Alzheimers Res Ther. 2024 May 11;16:106. doi: 10.1186/s13195-024-01476-x (PMC11088014; doi:10.1186/s13195-024-01476-x)
Supplement: Supplementary file 1 — Supplementary Material 1 [file 13195_2024_1476_MOESM1_ESM.doc]

**Supplemental materials**

**Table S1.** Codes for international classification disease for neurodegenerative outcomes.

**Table S2.** Genetic instrument for urate.

**Table S3.** Details of covariates.

**Table S4.** Association between urate levels and risk of neurodegenerative outcomes after excluding incidence in the first 5 years of follow-up.

**Table S5.** Association between urate levels and risk of neurodegenerative outcomes in subgroups.

**Table S6.** Association between urate levels and risk of neurodegenerative outcomes: results from competing risk regression model.

**Table S7.** Association between urate levels and the risk of ADRD and PD-related deaths.

**Table S8.** The casual associations between urate levels and neurodegenerative outcomes.

**Fig. S1** Flow chart for the selection of the analyzed study sample.

**Fig. S2** The distribution of urate-related GRS.

**Fig. S3** The casual associations between unweighted urate-related GRS and neurodegenerative outcomes using linear MR analysis.

**Fig. S4** The casual associations between rs2231142 and neurodegenerative outcomes using linear MR analysis.

Table S1. Codes for international classification disease for neurodegenerative outcomes.

| Outcome | ICD-9 | ICD-10 |
| --- | --- | --- |
| Alzheimer disease and related dementias | 331.0, 331.11, 331.19, 331.2, 331.7, 290.0, 290.10, 290.11, 290.12, 290.13, 290.20, 290.21, 290.3, 290.40, 290.41, 290.42, 290.43, 294.0, 294.10, 294.11, 294.20, 294.21, 294.8, 797 | F00, F00.0, F00.1, F00.2, F00.9, G30, G30.0, G30.1, G30.8, G30.9, F01, F01.0, F01.1, F01.2, F01.3, F01.8, F01.9, I67.3, F02.0, G31.0, A81.0, F02.1, F02.2, F02.3, F02.4, F10.6, F02, F02.8, F03, F05.1, G31.1, G31.8 |
| Parkinson | 332.0 332.1 | G20, G21, G22, G23， G259, G26, G903 |
| Neurodegenerative death |  | F00, F00.0, F00.1, F00.2, F00.9, G30, G30.0, G30.1, G30.8, G30.9, F01, F01.0, F01.1, F01.2, F01.3, F01.8, F01.9, I67.3, F02.0, G31.0, A81.0, F02.1, F02.2, F02.3, F02.4, F10.6, F02, F02.8, F03, F05.1, G31.1, G31.8, G20, G21, G22, G23， G259, G26, G903 |

Table S2. Genetic instrument for urate.

| SNPs | Chr. | Position | Closest gene | A2 | A1 | EAF | Beta | *P* value |
| --- | --- | --- | --- | --- | --- | --- | --- | --- |
| rs1471633 | 1 | 144435096 | PDZK1 | C | A | 0.46 | 0.059 | 1.20E-29 |
| rs11264341 | 1 | 153418117 | TRIM46 | C | T | 0.43 | -0.05 | 6.20E-19 |
| rs17050272 | 2 | 121022910 | INHBB | G | A | 0.43 | 0.035 | 1.60E-10 |
| rs2307394 | 2 | 148432898 | ORC4L | C | T | 0.68 | -0.029 | 2.20E-08 |
| rs6770152 | 3 | 53075254 | SFMBT1 | G | T | 0.58 | -0.044 | 2.60E-16 |
| rs12498742 | 4 | 9553150 | SLC2A9 | G | A | 0.77 | 0.373 | 1.00E-70 |
| rs2231142 | 4 | 89271347 | ABCG2 | G | T | 0.11 | 0.217 | 1.00E-134 |
| rs17632159 | 5 | 72467238 | TMEM171 | G | C | 0.31 | -0.039 | 3.50E-11 |
| rs1165151 | 6 | 25929595 | SLC17A1 | G | T | 0.47 | -0.091 | 7.00E-70 |
| rs1178977 | 7 | 72494985 | BAZ1B | G | A | 0.81 | 0.047 | 1.20E-12 |
| rs1171614 | 10 | 61139544 | SLC16A9 | C | T | 0.22 | -0.079 | 2.30E-28 |
| rs2078267 | 11 | 64090690 | SLC22A11 | C | T | 0.51 | -0.073 | 9.40E-38 |
| rs478607 | 11 | 64234639 | NRXN2 | G | A | 0.84 | -0.047 | 4.40E-11 |
| rs642803 | 11 | 65317196 | OVOL1 | C | T | 0.46 | -0.036 | 2.90E-13 |
| rs653178 | 12 | 110492139 | ATXN2 | C | T | 0.51 | -0.035 | 7.20E-12 |
| rs6598541 | 15 | 97088658 | IGF1R | G | A | 0.36 | 0.043 | 4.80E-15 |
| rs7193778 | 16 | 68121391 | NFAT5 | C | T | 0.86 | -0.046 | 8.20E-10 |
| rs7188445 | 16 | 78292488 | MAF | G | A | 0.33 | -0.032 | 1.60E-09 |
| rs7224610 | 17 | 50719787 | HLF | C | A | 0.58 | -0.042 | 5.40E-17 |
| rs164009 | 17 | 71795264 | QRICH2 | G | A | 0.61 | 0.028 | 1.60E-07 |

**Table S3. Details of covariates.**

| **Covariates** | **Field IDs** | **Description** | **Variable type** | **Variable classification** |
| --- | --- | --- | --- | --- |
| Age | 21022 | Age at recruitment | Continuous | - |
| Sex | 31 | Sex | Categorical | Female/Male |
| BMI | 21001 | Body mass index | Continuous | - |
| Education levels | 6138 | Qualifications | Categorical | College graduate or above/below |
| Townsend deprivation index | 22189 | Townsend deprivation index at recruitment | Continuous | - |
| Smoking status | 20116 | Smoking status | Categorical | Never/Previous/Current |
| Alcohol consumption | 1558 | Alcohol intake frequency | Categorical | Daily or almost daily/more than once a week/more than once a month/less than once a month/never |
| Family history of diseases | 20107/20110/20111 | Illnesses of father/mother/siblings | Categorical | Cardiovascular disease/hypertension/diabetes |
| Personal history of diseases | 20002 | Non-cancer illness code, self-reported | Categorical | Hypertension/diabetes/kidney disease/cardiovascular disease |
| Diet score |  |  | Categorical | The total diet score ranged from 0 to 7. |
| Fish | 1329/1339 | Oily/non-oily fish intake |
| Processed meats | 1349 | Processed meat |
| Unprocessed red meat | 1369/1379/1389 | Beef/lamb or mutton/pork |
| Fruits | 1309/1319 | Fresh/dried fruit intake |
| Vegetables | 1289/1299 | Cooked/salad /raw vegetable intake |
| Whole/refined grains | 1438/1448/1458/1468 | Wholemeal/wholegrain bread, bran/oat/muesli cereal intake |

**Table S4. Association between urate levels and risk of neurodegenerative outcomes after excluding incidence in the first 5 y**ears of follow-up.

|  | Quartiles of urate levels | | | | | *P* for trend ^1^ |
| --- | --- | --- | --- | --- | --- | --- |
|  | Quartile 1 | Quartile 2 | Quartile 3 | Quartile 4 |  | |
| Level of urate (umol/L): median | 218.9 | 260.9 | 304.7 | 403.5 |  | |
| Number of participants | 95,423 | 95,451 | 95,309 | 95,460 |  | |
| **Alzheimer and related dementia** |  |  |  |  |  | |
| Number of cases | 1,170 | 1,080 | 1,047 | 1,343 |  | |
| Person years | 1,105,125 | 1,108,419 | 1,107,140 | 1,104,547 |  | |
| Model 1 ^2^ | 1 (reference) | **0.84 (0.77, 0.91)** ^3^ | **0.74 (0.68, 0.81)** | **0.83 (0.76, 0.90)** | **<0.0001** | |
| Model 2 ^4^ | 1 (reference) | **0.86 (0.79, 0.93)** | **0.77 (0.71, 0.84)** | **0.86 (0.79, 0.94)** | **0.0002** | |
| Model 3 ^5^ | 1 (reference) | **0.88 (0.81, 0.96)** | **0.79 (0.73, 0.86)** | **0.86 (0.79, 0.93)** | **0.0001** | |
| **Parkinson** |  |  |  |  |  | |
| Number of cases | 502 | 463 | 469 | 474 |  | |
| Person years | 1,106,056 | 1,109,230 | 1,107,864 | 1,105,889 |  | |
| Model 1 | 1 (reference) | **0.86 (0.76, 0.98)** | **0.82 (0.72, 0.93)** | **0.75 (0.66, 0.86)** | **<0.0001** | |
| Model 2 | 1 (reference) | **0.87 (0.77, 0.99)** | **0.84 (0.74, 0.95)** | **0.77 (0.68, 0.88)** | **0.0002** | |
| Model 3 | 1 (reference) | 0.89 (0.78, 1.01) | **0.86 (0.75, 0.97)** | **0.78 (0.68, 0.89)** | **0.0004** | |
| **Neurodegenerative death** |  |  |  |  |  | |
| Number of cases | 318 | 272 | 277 | 300 |  | |
| Person years | 1,107,250 | 1,110,390 | 1,108,929 | 1,106,912 |  | |
| Model 1 | 1 (reference) | **0.79 (0.67, 0.93)** | **0.76 (0.64, 0.9)** | **0.74 (0.63, 0.88)** | **0.0007** | |
| Model 2 | 1 (reference) | **0.81 (0.69, 0.96)** | **0.79 (0.67, 0.93)** | **0.77 (0.65, 0.92)** | **0.004** | |
| Model 3 | 1 (reference) | **0.84 (0.71, 0.99)** | **0.81 (0.69, 0.96)** | **0.78 (0.66, 0.93)** | **0.006** | |

^1^ Analysis by Cox proportional hazards regression models.

^2^ Adjusted for age, sex, and BMI.

^3^ Hazard ratios (95% confidence interval) (all such values).

^4^ Additionally adjusted for education levels, Townsend deprivation index, smoking status, and alcohol consumption.

^5^ Additionally adjusted for family history of diseases (hypertension, cardiovascular disease, and diabetes), healthy diet score, and history of diseases (kidney disease, hypertension, cardiovascular disease, and diabetes).

**Table S5**. Association between urate levels and risk of neurodegenerative outcomes in subgroups.

| Subgroups | Models | Quartiles of urate levels | | | | *P* for trend ^1^ |
| --- | --- | --- | --- | --- | --- | --- |
|  |  | Quartile 1 | Quartile 2 | Quartile 3 | Quartile 4 |  |
| **Alzheimer and related dementia** |  |  |  |  |  |  |
| Age<60y | Number of cases | 193 | 160 | 160 | 146 |  |
|  | Number of participants | 57,665 | 54,428 | 51,343 | 46,159 |  |
|  | Model 1 ^2^ | 1 (reference) | **0.80 (0.65, 0.99)** ^3^ | **0.77 (0.62, 0.96)** | **0.69 (0.55, 0.87)** | **0.002** |
|  | Model 2 ^4^ | 1 (reference) | 0.83 (0.68, 1.03) | 0.82 (0.66, 1.02) | **0.74 (0.58, 0.93)** | **0.01** |
|  | Model 3 ^5^ | 1 (reference) | 0.88 (0.71, 1.09) | 0.88 (0.71, 1.09) | **0.76 (0.60, 0.96)** | **0.03** |
| Age≥60y | Number of cases | 1,197 | 1,091 | 1,068 | 1,385 |  |
|  | Number of participants | 37,938 | 41,147 | 44,093 | 49,409 |  |
|  | Model 1 | 1 (reference) | **0.82 (0.76, 0.89)** | **0.73 (0.68, 0.80)** | **0.82 (0.75, 0.89)** | **<0.0001** |
|  | Model 2 | 1 (reference) | **0.84 (0.78, 0.92)** | **0.76 (0.70, 0.83)** | **0.85 (0.78, 0.92)** | **<0.0001** |
|  | Model 3 | 1 (reference) | **0.86 (0.80, 0.94)** | **0.78 (0.72, 0.85)** | **0.84 (0.78, 0.92)** | **<0.0001** |
| Men | Number of cases | 817 | 672 | 631 | 686 |  |
|  | Number of participants | 43,768 | 43,795 | 43,675 | 43,752 |  |
|  | Model 1 | 1 (reference) | **0.80 (0.73, 0.89)** | **0.74 (0.66, 0.82)** | **0.76 (0.68, 0.84)** | **<0.0001** |
|  | Model 2 | 1 (reference) | **0.83 (0.75, 0.92)** | **0.77 (0.70, 0.86)** | **0.80 (0.72, 0.89)** | **<0.0001** |
|  | Model 3 | 1 (reference) | **0.86 (0.78, 0.96)** | **0.81 (0.73, 0.90)** | **0.82 (0.74, 0.91)** | **0.0002** |
| Women | Number of cases | 573 | 579 | 597 | 845 |  |
|  | Number of participants | 51,835 | 51,780 | 51,761 | 51,816 |  |
|  | Model 1 | 1 (reference) | **0.84 (0.75, 0.94)** | **0.75 (0.66, 0.84)** | **0.85 (0.76, 0.96)** | **0.007** |
|  | Model 2 | 1 (reference) | **0.86 (0.76, 0.96)** | **0.77 (0.68, 0.86)** | **0.88 (0.78, 0.98)** | **0.02** |
|  | Model 3 | 1 (reference) | **0.87 (0.77, 0.97)** | **0.77 (0.68, 0.87)** | **0.83 (0.74, 0.94)** | **0.001** |
| BMI<25 kg/m^2^ | Number of cases | 643 | 452 | 310 | 226 |  |
|  | Number of participants | 49,640 | 36,978 | 26,086 | 13,744 |  |
|  | Model 1 | 1 (reference) | **0.86 (0.77, 0.98)** | **0.76 (0.66, 0.88)** | 0.93 (0.80, 1.08) | **0.01** |
|  | Model 2 | 1 (reference) | 0.89 (0.79, 1.00) | **0.78 (0.68, 0.90)** | 0.95 (0.81, 1.11) | **0.04** |
|  | Model 3 | 1 (reference) | 0.90 (0.79, 1.01) | **0.78 (0.68, 0.89)** | 0.90 (0.77, 1.05) | **0.009** |
| BM≥25 kg/m^2^ | Number of cases | 747 | 799 | 918 | 1,305 |  |
|  | Number of participants | 45,963 | 58,597 | 69,350 | 81,824 |  |
|  | Model 1 | 1 (reference) | **0.82 (0.74, 0.91)** | **0.76 (0.69, 0.83)** | **0.79 (0.72, 0.87)** | **<0.0001** |
|  | Model 2 | 1 (reference) | **0.84 (0.76, 0.93)** | **0.79 (0.71, 0.87)** | **0.82 (0.75, 0.91)** | **0.0003** |
|  | Model 3 | 1 (reference) | **0.87 (0.79, 0.97)** | **0.82 (0.74, 0.90)** | **0.84 (0.76, 0.92)** | **0.0005** |
| **Parkinson** |  |  |  |  |  |  |
| Age<60y | Number of cases | 158 | 134 | 115 | 100 |  |
|  | Number of participants | 57,665 | 54,428 | 51,343 | 46,159 |  |
|  | Model 1 | 1 (reference) | 0.82 (0.65, 1.03) | **0.67 (0.53, 0.86)** | **0.57 (0.43, 0.74)** | **<0.0001** |
|  | Model 2 | 1 (reference) | 0.83 (0.66, 1.05) | **0.70 (0.54, 0.89)** | **0.60 (0.45, 0.78)** | **<0.0001** |
|  | Model 3 | 1 (reference) | 0.86 (0.68, 1.08) | **0.72 (0.56, 0.93)** | **0.62 (0.47, 0.81)** | **0.0002** |
| Age≥60y | Number of cases | 551 | 497 | 494 | 504 |  |
|  | Number of participants | 37,938 | 41,147 | 44,093 | 49,409 |  |
|  | Model 1 | 1 (reference) | **0.84 (0.74, 0.94)** | **0.78 (0.69, 0.88)** | **0.70 (0.62, 0.80)** | **<0.0001** |
|  | Model 2 | 1 (reference) | **0.85 (0.75, 0.96)** | **0.80 (0.70, 0.90)** | **0.73 (0.64, 0.83)** | **<0.0001** |
|  | Model 3 | 1 (reference) | **0.86 (0.76, 0.97)** | **0.81 (0.71, 0.91)** | **0.73 (0.64, 0.83)** | **<0.0001** |
| Men | Number of cases | 482 | 414 | 368 | 339 |  |
|  | Number of participants | 43,768 | 43,795 | 43,675 | 43,752 |  |
|  | Model 1 | 1 (reference) | **0.85 (0.74, 0.97)** | **0.74 (0.65, 0.85)** | **0.66 (0.57, 0.76)** | **<0.0001** |
|  | Model 2 | 1 (reference) | **0.86 (0.75, 0.98)** | **0.76 (0.66, 0.87)** | **0.68 (0.59, 0.79)** | **<0.0001** |
|  | Model 3 | 1 (reference) | 0.87 (0.76, 1.00) | **0.78 (0.68, 0.89)** | **0.70 (0.60, 0.81)** | **<0.0001** |
| Women | Number of cases | 227 | 217 | 241 | 265 |  |
|  | Number of participants | 51,835 | 51,780 | 51,761 | 51,816 |  |
|  | Model 1 | 1 (reference) | **0.81 (0.67, 0.97)** | **0.78 (0.65, 0.94)** | **0.69 (0.57, 0.84)** | **0.0004** |
|  | Model 2 | 1 (reference) | 0.83 (0.69, 1.00) | **0.81 (0.67, 0.98)** | **0.73 (0.60, 0.88)** | **0.002** |
|  | Model 3 | 1 (reference) | 0.83 (0.69, 1.00) | **0.81 (0.67, 0.97)** | **0.70 (0.58, 0.85)** | **0.0008** |
| BMI<25 kg/m^2^ | Number of cases | 308 | 200 | 119 | 88 |  |
|  | Number of participants | 49,640 | 36,978 | 26,086 | 13,744 |  |
|  | Model 1 | 1 (reference) | 0.83 (0.70, 1.00) | **0.66 (0.53, 0.82)** | 0.83 (0.65, 1.05) | **0.003** |
|  | Model 2 | 1 (reference) | 0.85 (0.71, 1.01) | **0.68 (0.54, 0.84)** | 0.86 (0.68, 1.10) | **0.009** |
|  | Model 3 | 1 (reference) | 0.85 (0.71, 1.01) | **0.67 (0.54, 0.83)** | 0.84 (0.66, 1.07) | **0.005** |
| BM≥25 kg/m^2^ | Number of cases | 401 | 431 | 490 | 516 |  |
|  | Number of participants | 45,963 | 58,597 | 69,350 | 81,824 |  |
|  | Model 1 | 1 (reference) | **0.85 (0.74, 0.97)** | **0.80 (0.70, 0.91)** | **0.66 (0.58, 0.75)** | **<0.0001** |
|  | Model 2 | 1 (reference) | **0.86 (0.75, 0.98)** | **0.81 (0.71, 0.93)** | **0.68 (0.59, 0.78)** | **<0.0001** |
|  | Model 3 | 1 (reference) | 0.87 (0.76, 1.00) | **0.83 (0.73, 0.95)** | **0.69 (0.60, 0.79)** | **<0.0001** |
| **Neurodegenerative death** |  |  |  |  |  |  |
| Age<60y | Number of cases | 43 | 39 | 30 | 23 |  |
|  | Number of participants | 57,665 | 54,428 | 51,343 | 46,159 |  |
|  | Model 1 | 1 (reference) | 0.93 (0.60, 1.44) | 0.72 (0.45, 1.17) | 0.61 (0.35, 1.04) | **0.04** |
|  | Model 2 | 1 (reference) | 0.95 (0.61, 1.47) | 0.75 (0.46, 1.21) | 0.64 (0.37, 1.11) | 0.08 |
|  | Model 3 | 1 (reference) | 0.95 (0.61, 1.48) | 0.75 (0.47, 1.22) | 0.64 (0.37, 1.10) | 0.07 |
| Age≥60y | Number of cases | 396 | 335 | 321 | 344 |  |
|  | Number of participants | 37,938 | 41,147 | 44,093 | 49,409 |  |
|  | Model 1 | 1 (reference) | **0.79 (0.68, 0.91)** | **0.71 (0.61, 0.83)** | **0.68 (0.59, 0.80)** | **<0.0001** |
|  | Model 2 | 1 (reference) | **0.80 (0.69, 0.93)** | **0.73 (0.63, 0.85)** | **0.71 (0.61, 0.83)** | **<0.0001** |
|  | Model 3 | 1 (reference) | **0.83 (0.71, 0.96)** | **0.76 (0.65, 0.88)** | **0.72 (0.62, 0.84)** | **<0.0001** |
| Men | Number of cases | 287 | 211 | 186 | 177 |  |
|  | Number of participants | 43,768 | 43,795 | 43,675 | 43,752 |  |
|  | Model 1 | 1 (reference) | **0.74 (0.62, 0.89)** | **0.66 (0.55, 0.80)** | **0.63 (0.52, 0.76)** | **<0.0001** |
|  | Model 2 | 1 (reference) | **0.76 (0.64, 0.91)** | **0.69 (0.57, 0.83)** | **0.65 (0.54, 0.79)** | **<0.0001** |
|  | Model 3 | 1 (reference) | **0.78 (0.66, 0.94)** | **0.71 (0.59, 0.86)** | **0.68 (0.56, 0.83)** | **<0.0001** |
| Women | Number of cases | 152 | 163 | 165 | 190 |  |
|  | Number of participants | 51,835 | 51,780 | 51,761 | 51,816 |  |
|  | Model 1 | 1 (reference) | 0.90 (0.72, 1.13) | 0.80 (0.64, 1.00) | **0.76 (0.61, 0.96)** | **0.01** |
|  | Model 2 | 1 (reference) | 0.92 (0.74, 1.15) | 0.83 (0.66, 1.04) | 0.79 (0.63, 1.00) | **0.03** |
|  | Model 3 | 1 (reference) | 0.94 (0.75, 1.17) | 0.83 (0.66, 1.04) | **0.76 (0.60, 0.96)** | **0.01** |
| BMI<25 kg/m^2^ | Number of cases | 214 | 148 | 102 | 62 |  |
|  | Number of participants | 49,640 | 36,978 | 26,086 | 13,744 |  |
|  | Model 1 | 1 (reference) | 0.87 (0.70, 1.08) | **0.78 (0.62, 0.99)** | 0.79 (0.59, 1.06) | **0.03** |
|  | Model 2 | 1 (reference) | 0.89 (0.72, 1.10) | 0.81 (0.63, 1.03) | 0.82 (0.61, 1.10) | 0.07 |
|  | Model 3 | 1 (reference) | 0.89 (0.72, 1.11) | 0.80 (0.63, 1.02) | 0.79 (0.59, 1.05) | **0.04** |
| BM≥25 kg/m^2^ | Number of cases | 225 | 226 | 249 | 305 |  |
|  | Number of participants | 45,963 | 58,597 | 69,350 | 81,824 |  |
|  | Model 1 | 1 (reference) | **0.78 (0.65, 0.94)** | **0.71 (0.59, 0.85)** | **0.65 (0.54, 0.78)** | **<0.0001** |
|  | Model 2 | 1 (reference) | **0.80 (0.66, 0.96)** | **0.73 (0.61, 0.87)** | **0.68 (0.57, 0.81)** | **<0.0001** |
|  | Model 3 | 1 (reference) | 0.83 (0.69, 1.00) | **0.76 (0.64, 0.92)** | **0.70 (0.58, 0.84)** | **0.0001** |

^1^ Analysis by Cox proportional hazards regression models.

^2^ Adjusted for age, sex, and BMI.

^3^ Hazard ratios (95% confidence interval) (all such values).

^4^ Additionally adjusted for education levels, Townsend deprivation index, smoking status, and alcohol consumption.

^5^ Additionally adjusted for family history of diseases (hypertension, cardiovascular disease, and diabetes), healthy diet score, and history of diseases (kidney disease, hypertension, cardiovascular disease, and diabetes).

**Table S6**. Association between urate levels and risk of neurodegenerative outcomes: results from competing risk regression model.

|  | Quartiles of urate levels | | | |
| --- | --- | --- | --- | --- |
|  | Quartile 1 | Quartile 2 | Quartile 3 | Quartile 4 |
| **Alzheimer and related dementia** |  |  |  |  |
| Model 1 ^2^ | 1 (reference) | **0.82 (0.76, 0.89)** ^3^ | **0.74 (0.69, 0.80)** | **0.80 (0.74, 0.86)** |
| Model 2 ^4^ | 1 (reference) | **0.85 (0.78, 0.91)** | **0.77 (0.71, 0.84)** | **0.83 (0.77, 0.90)** |
| Model 3 ^5^ | 1 (reference) | **0.87 (0.81, 0.94)** | **0.79 (0.73, 0.86)** | **0.83 (0.76, 0.89)** |
| **Parkinson** |  |  |  |  |
| Model 1 | 1 (reference) | **0.84 (0.75, 0.93)** | **0.76 (0.68, 0.85)** | **0.67 (0.60, 0.76)** |
| Model 2 | 1 (reference) | **0.85 (0.76, 0.95)** | **0.78 (0.70, 0.87)** | **0.70 (0.62, 0.78)** |
| Model 3 | 1 (reference) | **0.86 (0.77, 0.96)** | **0.79 (0.71, 0.88)** | **0.70 (0.62, 0.79)** |
| **Neurodegenerative death** |  |  |  |  |
| Model 1 | 1 (reference) | **0.80 (0.70, 0.92)** | **0.71 (0.62, 0.82)** | **0.67 (0.58, 0.78)** |
| Model 2 | 1 (reference) | **0.82 (0.71, 0.94)** | **0.74 (0.64, 0.85)** | **0.70 (0.60, 0.81)** |
| Model 3 | 1 (reference) | **0.84 (0.73, 0.97)** | **0.76 (0.66, 0.88)** | **0.71 (0.61, 0.82)** |

^1^ Analysis by Cox proportional hazards regression models.

^2^ Adjusted for age, sex, and BMI.

^3^ Hazard ratios (95% confidence interval) (all such values).

^4^ Additionally adjusted for education levels, Townsend deprivation index, smoking status, and alcohol consumption.

^5^ Additionally adjusted for family history of diseases (hypertension, cardiovascular disease, and diabetes), healthy diet score, and history of diseases (kidney disease, hypertension, cardiovascular disease, and diabetes).

**Table S7. Association between urate levels and the risk of ADRD and PD-related deaths.**

|  | Quartiles of urate levels | | | | *P* for trend ^1^ |
| --- | --- | --- | --- | --- | --- |
|  | Quartile 1 | Quartile 2 | Quartile 3 | Quartile 4 |  |
| Number of participants | 95,603 | 95,575 | 95,436 | 95,568 |  |
| **ADRD-related death** |  |  |  |  |  |
| Number of cases | 343 | 293 | 278 | 321 |  |
| Person years | 1,138,283 | 1,138,755 | 1,138,978 | 1,146,720 |  |
| Model 1 ^2^ | 1 (reference) | **0.80 (0.68, 0.94)** ^3^ | **0.72 (0.61, 0.84)** | **0.74 (0.63, 0.87)** | **0.0001** |
| Model 2 ^4^ | 1 (reference) | **0.82 (0.70, 0.96)** | **0.74 (0.63, 0.87)** | **0.76 (0.65, 0.90)** | **0.0007** |
| Model 3 ^5^ | 1 (reference) | **0.85 (0.72, 0.99)** | **0.77 (0.65, 0.90)** | **0.78 (0.66, 0.92)** | **0.001** |
| **PD-related death** |  |  |  |  |  |
| Number of cases | 96 | 81 | 73 | 46 |  |
| Person years | 1,138,939 | 1,139,292 | 1,139,422 | 1,147,261 |  |
| Model 1 | 1 (reference) | 0.82 (0.61, 1.11) | **0.71 (0.52, 0.98)** | **0.42 (0.29, 0.60)** | **<0.0001** |
| Model 2 | 1 (reference) | 0.84 (0.62, 1.13) | 0.74 (0.54, 1.01) | **0.44 (0.30, 0.64)** | **<0.0001** |
| Model 3 | 1 (reference) | 0.84 (0.62, 1.14) | 0.74 (0.54, 1.01) | **0.42 (0.29, 0.61)** | **<0.0001** |

Abbreviations: ADRD, Alzheimer and related dementia; PD, Parkinson.

^1^ Analysis by Cox proportional hazards regression models.

^2^ Adjusted for age, sex, and BMI.

^3^ Hazard ratios (95% confidence interval) (all such values).

^4^ Additionally adjusted for education levels, Townsend deprivation index, smoking status, and alcohol consumption.

^5^ Additionally adjusted for family history of diseases (hypertension, cardiovascular disease, and diabetes), healthy diet score, and history of diseases (kidney disease, hypertension, cardiovascular disease, and diabetes).

**Table S8**. The casual associations between urate levels and neurodegenerative outcomes.

|  | Urate-related GRS | | | | *P* for trend ^1^ |
| --- | --- | --- | --- | --- | --- |
|  | Quartile 1 | Quartile 2 | Quartile 3 | Quartile 4 |  |
| Number of participants | 98,397 | 96,315 | 91,002 | 96,468 |  |
| **Alzheimer and related dementia** |  |  |  |  |  |
| Number of cases | 1,419 | 1,359 | 1,277 | 1,345 |  |
| Person years | 1,419 | 1,359 | 1,277 | 1,345 |  |
| Model 1 ^2^ | 1,141,942 | 1,117,001 | 1,055,482 | 1,119,038 |  |
| Model 2 ^4^ | 1 (reference) | 0.97 (0.90, 1.05) ^3^ | 0.96 (0.89, 1.04) | 0.96 (0.89, 1.03) | 0.32 |
| Model 3 ^5^ | 1 (reference) | 0.97 (0.90, 1.05) | 0.97 (0.89, 1.04) | 0.96 (0.89, 1.04) | 0.35 |
| **Parkinson** | 1 (reference) | 0.97 (0.90, 1.05) | 0.97 (0.89, 1.04) | 0.96 (0.89, 1.04) | 0.33 |
| Number of cases |  |  |  |  |  |
| Person years | 616 | 658 | 601 | 678 |  |
| Model 1 | 1,142,813 | 1,117,666 | 1,056,200 | 1,119,599 |  |
| Model 2 | 1 (reference) | 1.08 (0.97, 1.21) | 1.05 (0.94, 1.17) | 1.12 (1.00, 1.25) | 0.07 |
| Model 3 | 1 (reference) | 1.08 (0.97, 1.21) | 1.05 (0.94, 1.17) | 1.12 (1.00, 1.25) | 0.07 |
| **Neurodegenerative death** | 1 (reference) | 1.08 (0.97, 1.21) | 1.05 (0.94, 1.17) | 1.12 (1.00, 1.25) | 0.07 |
| Number of cases |  |  |  |  |  |
| Person years | 382 | 399 | 358 | 392 |  |
| Model 1 | 1,144,930 | 1,119,923 | 1,058,331 | 1,121,947 |  |
| Model 2 | 1 (reference) | 1.06 (0.92, 1.22) | 1.00 (0.87, 1.16) | 1.04 (0.90, 1.20) | 0.73 |
| Model 3 | 1 (reference) | 1.06 (0.92, 1.22) | 1.01 (0.87, 1.16) | 1.04 (0.90, 1.20) | 0.71 |

^1^ Analysis by Cox proportional hazards regression models.

^2^ Adjusted for age, sex, and BMI.

^3^ Hazard ratios (95% confidence interval) (all such values).

^4^ Additionally adjusted for education levels, Townsend deprivation index, smoking status, and alcohol consumption.

^5^ Additionally adjusted for family history of diseases (hypertension, cardiovascular disease, and diabetes), healthy diet score, history of diseases (kidney disease, hypertension, cardiovascular disease, and diabetes), first 10 principal components of ancestry, and genotype measurement batch.

502,389 participants who provided informed consent at baseline

382,614 follow participants

119,775 excluded

33,653 with missing data on urate levels

5,173 with missing data on genetic data

343 with sex discordance, or outliers with genotype missingness or heterozygosity

74,248 related individuals, or individuals of non-European ancestry

280 with prevalent ADRD or PD at the baseline

6,078 with missing data on covariates

432 loss to follow-up

382,182 participants included in analysis

**Fig. S1** Flow chart for the selection of the analyzed study sample.

**Fig. S2** The distribution of urate-related GRS.


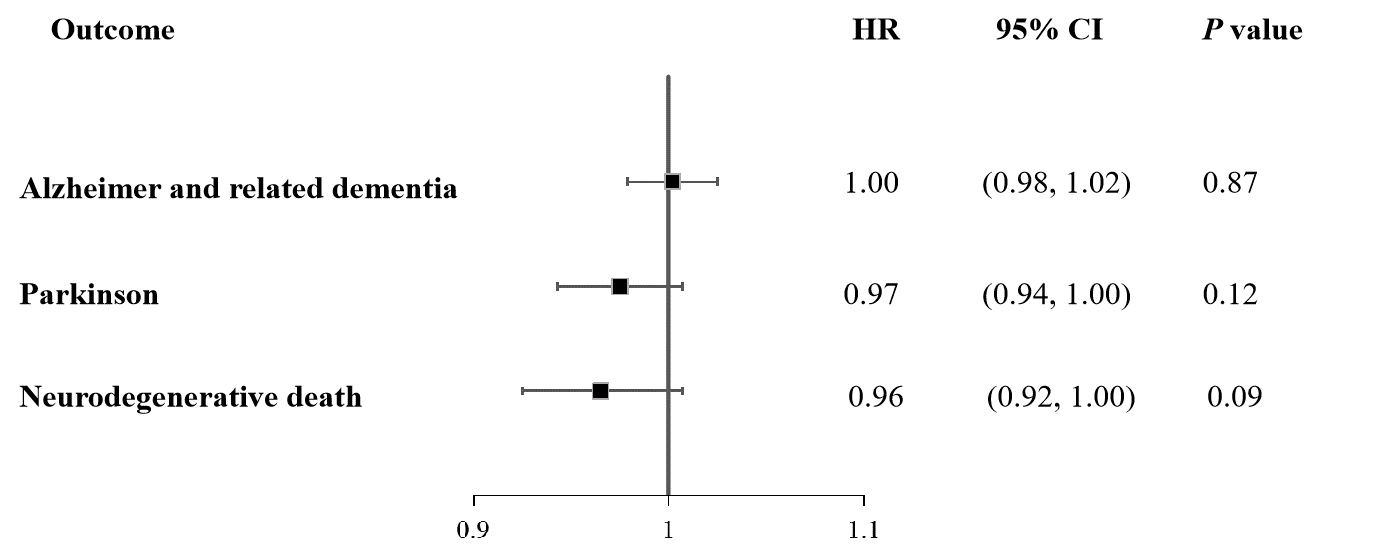


**Fig. S3** The casual associations between unweighted urate-related GRS and neurodegenerative outcomes using linear MR analysis. Adjusted for age, sex, BMI, education levels, Townsend deprivation index, smoking status, alcohol consumption, family history of diseases (hypertension, cardiovascular disease, and diabetes), healthy diet score, history of diseases (kidney disease, hypertension, cardiovascular disease, and diabetes), first 10 principal components of ancestry, and genotype measurement batch.


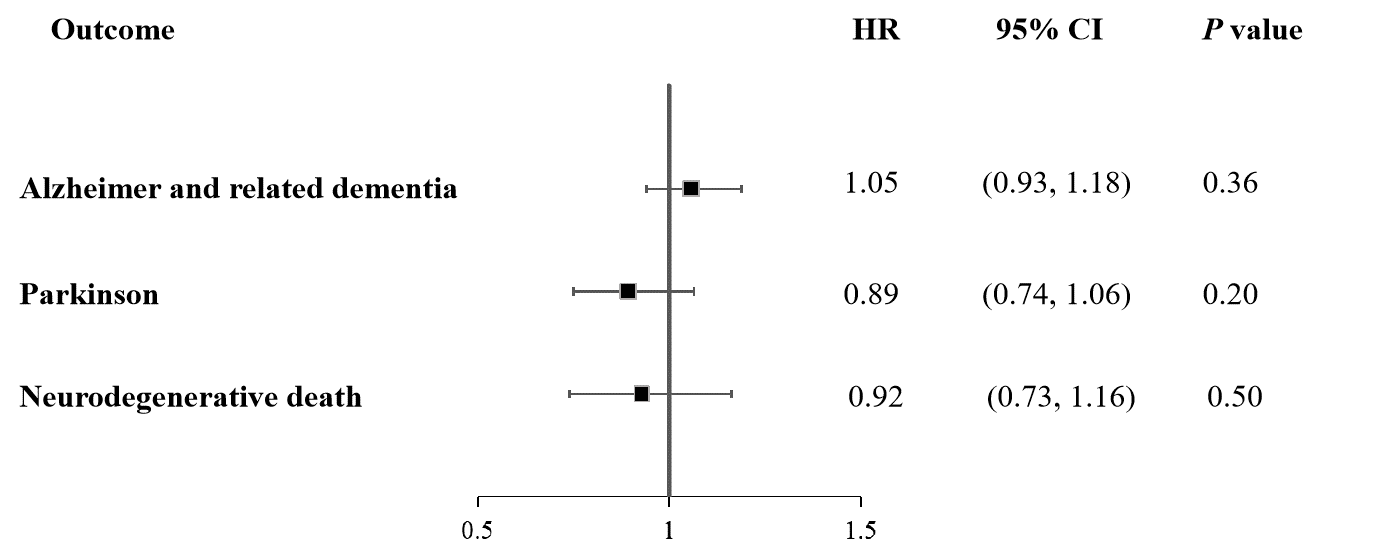


**Fig. S4** The casual associations between rs2231142 and neurodegenerative outcomes using linear MR analysis. Adjusted for age, sex, BMI, education levels, Townsend deprivation index, smoking status, alcohol consumption, family history of diseases (hypertension, cardiovascular disease, and diabetes), healthy diet score, history of diseases (kidney disease, hypertension, cardiovascular disease, and diabetes), first 10 principal components of ancestry, and genotype measurement batch.
